# Direct rapid antimicrobial susceptibility testing (RAST) from positive blood cultures: Recent advances, challenges, and future directions

**DOI:** 10.1016/j.plabm.2026.e00555

**Published:** 2026-09-04

**Authors:** Haiyi Li, Qiongfang Zhu, Yalu Ren, Meili Shen, Jie Xu

**Affiliations:** a Center of Clinical Laboratory, The First Affiliated Hospital of Soochow University, Suzhou, China; b MOE Key Laboratory of Geriatric Diseases and Immunology, Suzhou Key Laboratory of Pathogen Bioscience and Anti-infective Medicine, Soochow University, Suzhou, China; c Medical Department, Nanjing Dinfectome Technology Inc., Nanjing, China

**Keywords:** Bloodstream infections, Positive blood cultures, Rapid antimicrobial susceptibility testing, Performance, Limitations

## Abstract

Bloodstream infections (BSIs) carry high morbidity and mortality, and delayed targeted therapy worsens survival. Conventional antimicrobial susceptibility testing (AST) from positive blood cultures typically requires 24–48 h (and >72 h for some resistant organisms), delaying appropriate treatment and leaving 14–78% of empirical regimens unnecessary or ineffective. Direct rapid antimicrobial susceptibility testing (RAST) addresses this bottleneck by delivering AST in 4–16 h with categorical agreement (CA) ≥90% for major pathogens, using volatile organic compound (VOC) detection, modified disk diffusion, microfluidics, and molecular approaches. RAST generally makes susceptibility results available approximately 8–44 h earlier, with savings exceeding 56 h when conventional testing requires more than 72 h. This review compares the principles, turnaround times (TAT), and performance of leading RAST platforms. It further identifies persistent bottlenecks restricting cross-platform comparability and clinical uptake, most notably the absence of standardized protocols, the inability of molecular assays to determine minimum inhibitory concentrations (MICs), and unresolved pre-analytical confounders. Although RAST reliably shortens time-to-result and supports antimicrobial stewardship, its routine integration demands platform-specific validation, transparent regulatory and cost-effectiveness assessment.

## Highlights

• RAST shortens blood-culture AST turnaround time to 4–16 h (some platforms <3 h).
• VOC, modified disk-diffusion, and microfluidic RAST achieve ≥90% CA for major BSI pathogens.
• Molecular RAST detects resistance genes in 1–3 h but cannot generate MIC values, creating genotype–phenotype discordance risk.
• Lack of standardized protocols and unaddressed pre-analytical variables remain the key barriers to cross-platform comparability.

## 1 Introduction

Bloodstream infections (BSIs) are among the leading causes of death in critically ill patients worldwide. More than 40 million sepsis cases occur globally each year, with mortality ranging from 20% to 50% [1]. The annual economic burden exceeds US$25 billion in the United States alone [2]. Timely initiation of targeted antimicrobial therapy is essential to reduce mortality and improve outcomes in BSIs, and each hour of delay in effective treatment is associated with a significant survival penalty in septic shock [[3], [4], [5]].

**Fig. 1:**
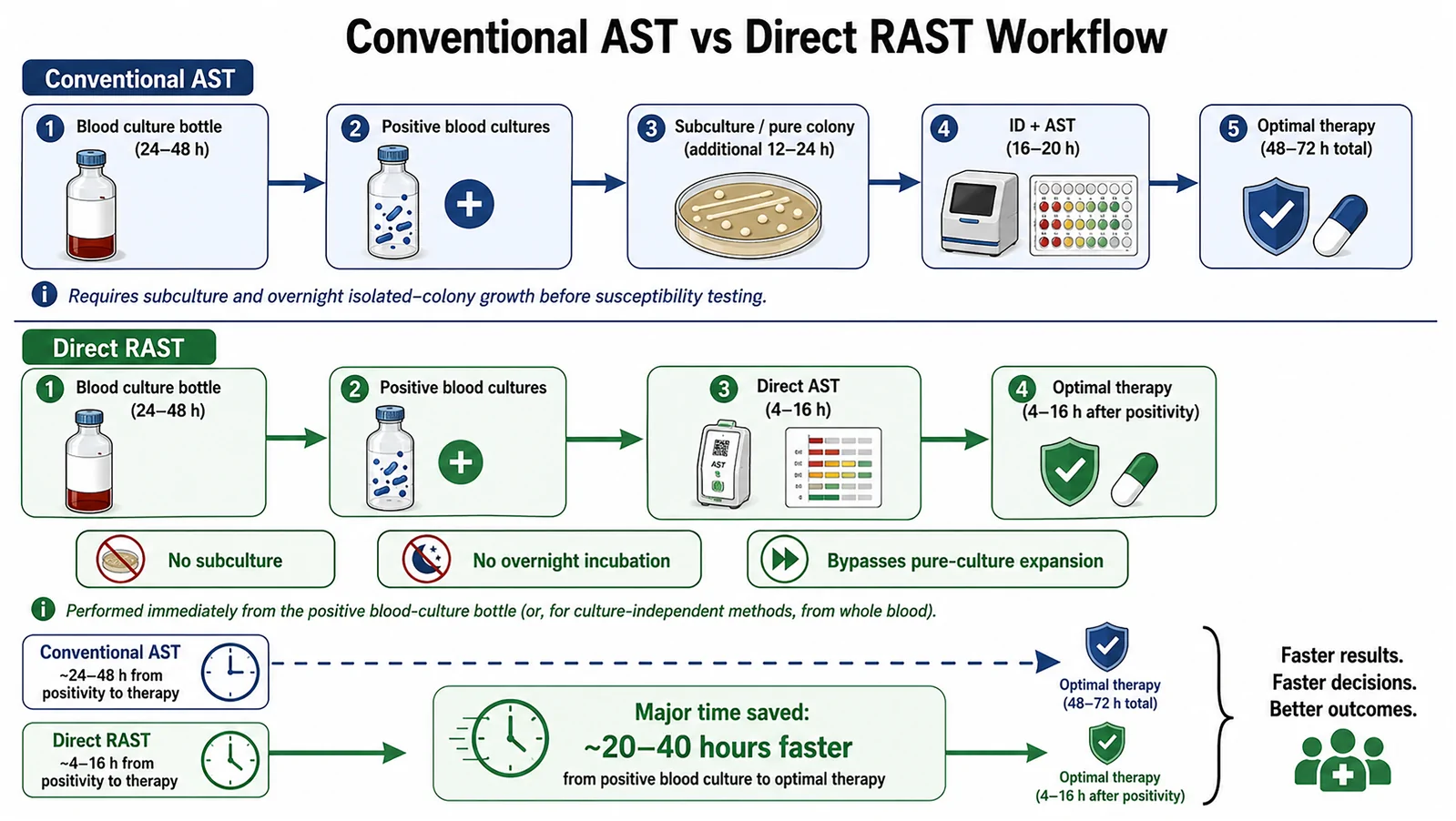
Conventional AST vs Direct RAST Workflow.

Conventional antimicrobial susceptibility testing (AST) applied to positive blood cultures, including colony isolation and purification, inoculation, and result interpretation, typically requires a total turnaround time (TAT) of 24 to 48 h, and detection of certain resistant strains may exceed 72 h [6,7]. With the continuing rise of antimicrobial resistance (AMR), the proliferation of multidrug-resistant (MDR) and extensively drug-resistant (XDR) strains further complicates empirical therapy. Approximately 16% to 79% of empirically prescribed antibiotics are unnecessary or ineffective [[8], [9], [10], [11], [12]], which not only delays appropriate treatment but also promotes resistance transmission and adverse events [13]. Rapid antimicrobial susceptibility testing (RAST) analyzes positive blood cultures directly, bypassing colony purification, and employs innovative detection methods to shorten incubation, delivering susceptibility results within 4–16 h; some technologies achieve results in only a few hours (Fig. 1) [2,14].

In recent years, multiple RAST platforms have been developed and clinically evaluated, including VITEK® REVEAL™, EUCAST RAST, Accelerate PhenoTest, and uRAST. Their principles span phenotypic detection (e.g., metabolite monitoring and growth-kinetic analysis) and genotypic detection (e.g., rapid amplification of resistance genes), with phenotypic detection remaining predominant because it directly reflects the actual susceptibility of clinical strains and better matches prescribing needs [2,15].

This review focuses on methods that perform AST directly from positive blood-culture bottles. To keep the comparison coherent, we prioritized platforms with published, peer-reviewed performance data from positive blood cultures and clear routes to clinical implementation. Established automated systems that still require isolated colonies or prolonged incubation before RAST (e.g., BD Phoenix, MALDI-TOF VITEK MS used for identification rather than direct RAST) and very early-stage technologies lacking head-to-head validation (e.g., large-volume scattering imaging, nascent AI-driven systems) are accordingly outside the core scope. We additionally include culture-independent microfluidic approaches (e.g., uRAST) as an emerging adjacent category, because they target the same BSI diagnostic bottleneck and inform the future trajectory of direct testing. The uRAST platform captures bacteria directly from whole blood rather than from an already-positive culture bottle. It is included here not as a conventional positive-bottle RAST, but as a representative of the broader “direct-from-specimen” paradigm that shares the same goal of circumventing culture amplification. This scope extension is made explicit so that readers interpret its performance relative to true positive-bottle methods. Throughout this review, agreement with reference methods is reported using standard metrics: categorical agreement (CA) is the proportion of isolate–drug results assigned to the same category (susceptible/intermediate/resistant, S/I/R) as the reference; essential agreement (EA) is the proportion of results within ±1 doubling dilution of the reference MIC; a very major error (VME) is the dangerous misclassification of a resistant strain as susceptible; and a major error (ME) is the misclassification of a susceptible strain as resistant.

This review explores the principles, performance profiles, clinical utility, and prevailing challenges and limitations of mainstream RAST technologies for positive blood cultures (Table 1).

**Table 1:** Comparison of different technologies of rapid antimicrobial susceptibility testing (RAST).

Platform	Principle	TAT	Validated Pathogens	Performance (vs reference)	Limitations	Future Directions
VITEK REVEAL	VOC sensing → MIC/S-I-R	3–8 h	*Enterobacteriaceae*, *P. aeruginosa*, *A. baumannii*, Gram-positive bacteria	CA ≥97.6% (*Enterobacteriaceae*), 97.9% (*P. aeruginosa*); NDM CA 63–100%	High ME for some drugs (TMP-SMX 56.3%); instrument cost	Expand agents; automated interpretation
EUCAST RAST	Modified disk diffusion	4–8 h	Mainly *Enterobacteriaceae*	CA 97–99% (4 h); 100% ESBL (6 h)	ATU deferrals; labor if not automated	MLA integration; extended incubation
Accelerate PhenoTest BC	Microfluidics + morphokinetics	∼7 h	Gram-negative bacteria + Gram-positive bacteria	CA 94.3% (Gram-negative rods)/97.9% (Gram-positive cocci); EA 95.4%/97.6%; VME 0.5%/1.0%	High capital cost; dedicated instrument	Broader panels
uRAST	Microfluidic capture from whole blood	13 ± 2.53 h	*E. coli*, *S. aureus* (capture 91.5–96.2%)	CA 94.90% (clinical); 95% (preclinical)	Culture-independent (not from bottle); research stage	Pre-culture-free direct testing
QuickMIC	Microfluidic + solid-phase cytometry → MIC	2–4 h (TTR 3 h 44 min)	Gram-negative bacteria	CA 86.2% (carbapenem-resistant Gram-negative isolates); EA 92.3% (*Enterobacteriaceae*)/97.0% (non-fermenters); VMD 0.7%	Meropenem “I” reporting bias (72.2%); cost	Faster MIC; wider species
BioFire BCID2	Multiplex PCR (resistance genes)	∼1 h	14 Gram-negative bacteria (gene targets)	Gene detection sensitivity ∼94%; no MIC	Genotype–phenotype discordance	Link to phenotypic confirmation
Verigene BC Test	DNA microarray (resistance genes)	∼2.5 h	9 Gram-negative bacteria (gene targets)	Gene detection sensitivity 89–100%; no MIC	No MIC; discordance risk	Multiplex expansion

Note:AST, antimicrobial susceptibility testing; ATU, area of technical uncertainty; BMD, broth microdilution; CA, categorical agreement; EA, essential agreement; ME, major error; MIC, minimum inhibitory concentration; NPA, negative percent agreement; PPA, positive percent agreement; TAT, turnaround time; TTR, time to result; VME, very major error. For molecular platforms, performance metrics refer to organism and resistance-gene detection rather than phenotypic AST agreement.

## 2 RAST platforms

### 2.1 Volatile organic compound (VOC) detection-based RAST

Bacteria synthesize distinct volatile organic compounds (VOCs) during growth, and the composition and concentration of these metabolites closely track metabolic state. Upon antimicrobial exposure, susceptible strains show growth inhibition with markedly reduced VOC release, whereas resistant strains maintain normal metabolism and an unchanged VOC signature [13]. The most representative platform is the VITEK® REVEAL™ system (bioMérieux), formerly SPECIFIC REVEAL®, which has obtained CE-IVD certification and FDA 510(k) clearance and is commercially available [13]. The system inoculates positive blood culture material onto antimicrobial-containing assay film; a colorimetric sensor array detects VOCs released during growth, and software analyzes color changes to automatically generate MIC values and S/I/R categories. The workflow requires minimal sample preparation, with hands-on time under 5 min and an incubation of typically 3–8 h (Table 1) [13,16].

For *Enterobacteriaceae* detection, the system demonstrated a categorical agreement (CA) consistently above 94% and an essential agreement (EA) exceeding 97% compared to traditional methods such as VITEK 2, broth microdilution (BMD), and Etest [13,17]. Bianco et al. [13] assessed VITEK® REVEAL™ using 128 Gram-negative isolates, including 95 *Enterobacteriaceae*, 21 *Pseudomonas aeruginosa*, and 12 *Acinetobacter baumannii* strains. Comparing results with reference methods (MicroScan Walkaway/ETEST/BMD), the system achieved an overall CA of 97.6% and EA of 97.7%, with corresponding values of 97.5% (CA) and 98.1% (EA) for *Enterobacteriaceae*. The very major error (VME), major error (ME), and minor error rates for *Enterobacteriaceae* were 1.6%, 1.4%, and 2.1%, respectively, all meeting CLSI-recommended standards (VME ≤1.5%, ME ≤ 3%) [13]. In a dedicated study on NDM-producing *Enterobacteriaceae* and NDM-producing *A. baumannii* [3], VITEK® REVEAL™ achieved CA values ranging from 63% to 100% for the detection of NDM-producing *Enterobacteriaceae* [13]. Notably, the VME rate for meropenem was 8%, while the ME rate for co-trimoxazole (trimethoprim–sulfamethoxazole) reached 56.3%, indicating that *Enterobacteriaceae* were the primary source of errors in carbapenem and aztreonam testing [16]. For NDM-producing *A. baumannii*, minor error rates for imipenem and meropenem were 35% and 76%, respectively [3]. When testing non-fermenting bacteria, VITEK® REVEAL™ achieved a CA of 97.9% and an EA of 95.2% for *P. aeruginosa*, and a CA of 97.9% for *A. baumannii* [13]. The system yielded CA values ranging from 98.6% to 100% for novel antimicrobials such as ceftazidime/avibactam and ceftolozane/tazobactam, providing rapid guidance for treating multidrug-resistant infections [17]. Furthermore, the VITEK® REVEAL™ system demonstrated excellent performance in detecting extended-spectrum β-lactamase (ESBL)-producing strains, achieving CA rates of 96.5% to 100% compared to reference methods [13,17].

As a near-fully automated, standardized assay, VITEK REVEAL is well suited to high-throughput laboratories, though it requires dedicated instrumentation and carries a higher capital cost than manual methods.

### 2.2 Modified disk diffusion-based RAST

In 2019, the European Committee on Antimicrobial Susceptibility Testing (EUCAST) published a protocol for rapid antimicrobial susceptibility testing (RAST) directly from positive blood cultures using the disk diffusion (DD) method [18]. Key technical improvements of EUCAST RAST comprise four components (Table 1) [[18], [19], [20]]. (1) Inoculum optimization: 100–150 μL of blood-culture broth is directly spread evenly onto Mueller–Hinton (MH) agar plates. (2) Standardized incubation: plates are incubated at 35°C under aerobic conditions. (3) Area of Technical Uncertainty (ATU): zone diameters at or near the susceptible/resistant (S/R) breakpoint are deferred and classified as ATU rather than misclassified, thereby reducing misclassification [6,21]. (4) Automation: integration with microbiological laboratory automation (MLA) systems such as WASPLab enables fully automated plate incubation, image capture, and zone measurement, enhancing both accuracy and efficiency [21].

In *Enterobacteriaceae* susceptibility testing, the 4-h EUCAST RAST achieved an overall CA of 97%–99% for *Escherichia coli* and *Klebsiella pneumoniae*, with VME rates of 0.4%–1.0% and ME rates of 0%–0.6% [6,10,19]. Analysis of 530 *E. coli* and 112 *K. pneumoniae* isolates demonstrated that at 4 h, CA exceeded 97% for cefotaxime, ceftazidime, meropenem, and ciprofloxacin, with meropenem reaching 99.8% [6]. The proportion of results classifiable as susceptible or resistant (S/R) ranged from 83.1% to 87.5%. Only piperacillin–tazobactam showed a lower classifiable rate (37.2% for *E. coli* and 66.1% for *K. pneumoniae*), primarily because a large number of results fell into the ATU category [6]. A multicenter study further demonstrated that the 4-, 6-, and 8-h EUCAST RAST achieved 100% CA for cefotaxime, detecting all 48 *E. coli* and *K. pneumoniae* isolates [10].

EUCAST RAST retains the simplicity and low consumable cost of conventional disk diffusion, making it attractive for resource-limited settings; however, manual reading at strict time points is labor-intensive, and full automation depends on MLA infrastructure.

### 2.3 Microfluidic and single-cell analysis-based RAST

The Accelerate Pheno system (Accelerate Diagnostics, USA) was the first technology commercially available in the United States to provide AST results directly from positive blood cultures, receiving initial FDA approval in 2017 [22]. It uses morphokinetic cellular analysis: a single 0.5 mL aliquot of positive blood cultures is loaded within 30 min of positivity, organism identification is performed by automated fluorescence in situ hybridization (FISH) at ∼2 h, and MIC is determined via morphokinetic growth analysis at ∼7 h [22]. In a multicenter evaluation, the system achieved CA of 97.9% for Gram-positive cocci and 94.3% for Gram-negative rods, with EA 97.6% and 95.4%, ME 0.7% and 0.9%, and VME 1.0% and 0.5%, respectively (Table 1) [22]. Accelerate Pheno is unique in performing identification in addition to AST from a single positive-bottle aliquot, whereas other direct-from-bottle systems require a separate identification step.

More recently, QuickMIC (Gradientech AB, Sweden) entered the market, combining a microfluidic and solid-phase cytometry approach [23,24]. It delivers automated phenotypic MIC results for 12 antibiotics in 2–4 h directly from a positive blood culture bottle [23,24]. In a head-to-head study of 101 carbapenem-resistant Gram-negative isolates, mean time to result (TTR) was 3 h 44 min, overall CA 86.2% versus BMD, and EA 92.3% for *Enterobacteriaceae* and 97.0% for non-fermenters, with VMD 0.7% [24]. QuickMIC generated 24.2% more valid results at the 4-h time point than EUCAST RAST, but it exhibited a negative reporting bias for meropenem: 72.2% of meropenem minor discrepancies in *Enterobacterales* were reported as “I” (susceptible, increased exposure) while BMD called “R” [24]. The resistance determinant most associated with QuickMIC errors was *NDM-5* carriage [24].

The uRAST platform represents a culture-independent, blood-culture-free approach. It uses sβ2GPI-peptide-coated magnetic nanoparticles to selectively capture a broad range of pathogens directly from whole blood, followed by species identification and a low-inoculum phenotypic AST chip [2]. In a clinical validation of 190 hospitalized, infection-suspected patients, species identification matched 100%, and among positive cases the AST showed overall categorical agreement of 94.90% with an average theoretical TAT of 13 ± 2.53 h from initial blood processing, reducing reporting time by an estimated 40–60 h versus hospital AST workflows (Table 1) [2]. Because it bypasses culture amplification entirely, uRAST is positioned here as a culture-independent member of the direct-testing paradigm and remains primarily at the research/validation stage [2].

### 2.4 Molecular diagnostics-based RAST

Molecular RAST detects resistance genes directly from positive blood cultures using multiplex nucleic-acid amplification, delivering results in 1–3 h without requiring isolated colonies [25,26].

The BioFire BCID2 (bioMérieux) is a multiplex PCR panel. Covering 14 Gram-negative targets, it reports resistance genes including *CTX-M* (ESBL), *IMP*, *KPC*, *OXA-48-like*, *NDM*, and *VIM* (carbapenemases), as well as *mcr-1* (colistin). The assay runs in approximately 1 h directly from the blood-culture bottle, with an overall sensitivity of about 94% [25]. More comprehensively, the BIOFIRE® BCID2 Panel detects 43 targets in total, comprising 14 Gram-negative bacteria, 10 Gram-positive bacteria, 7 fungi, and 12 resistance genes (e.g., *CTX-M, KPC, NDM, OXA-48*), with a TAT of just 1 h [27,28]. In terms of resistance-gene detection accuracy, the assay exhibits a sensitivity and specificity of over 95% for common resistance genes such as *CTX-M, KPC*, and *NDM* (Table 1) [27,28]. Because BioFire BCID2 reports resistance determinants rather than phenotypes, CA, EA, VME, ME, and MIC are not applicable to this platform. Resistance-gene detection sensitivity of ∼94% (and >95% for *CTX-M, KPC, NDM*) is the quantitative performance metric reported to date.

The Verigene BC Test (Luminex) is a DNA-microarray-based panel covering 9 Gram-negative resistance-gene targets (*CTX-M; KPC, NDM, VIM, IMP, OXA-48, OXA-23, OXA-24/40, OXA-58*) [26]. The assay runs in approximately 2.5 h directly from the blood-culture bottle, with a sensitivity of 89–100% (Table 1). More broadly, the Verigene BC Test directly detects Gram-positive bacteria, Gram-negative bacteria, and resistance genes (e.g., *mecA*, *vanA/B*) from positive blood cultures, with a TAT of approximately 2 h [4,29]. It delivers pathogen identification results 24 to 48 h earlier than conventional methods. As a gene-based panel, Verigene BC Test generates no phenotypic CA/EA or MIC. Its reported quantitative performance is therefore limited to resistance-gene detection sensitivity of 89–100%.

Currently, the greatest methodological limitation of molecular RAST is the discordance between resistance genotype and resistance phenotype. A critical clinical caveat is that molecular platforms report resistance determinants, not phenotypic MICs, and a detected gene does not always correspond to a resistant phenotype. Phenotypic assays remain the gold standard, and discordance between genotypic and phenotypic tests is occasionally observed, with interpretation not always straightforward [26]. Silent or variably expressed resistance genes, plasmid loss or acquisition after sampling, and sequence polymorphisms can all decouple genotype from phenotype. Therefore, molecular results frequently require phenotypic confirmation, particularly when (i) the detected gene is known for variable expression, (ii) local epidemiology suggests heteroresistance, or (iii) the result would drive de-escalation or avoidance of a last-line agent. Phenotypic RAST or conventional AST remains the confirmatory standard in these scenarios [26].

## 3 Technical challenges and limitations

### 3.1 Lack of standardization as a key barrier

The differences in detection principles and quality-control strategies across platforms are real, but their broader significance is that they reflect a lack of standardization that is itself the principal barrier to comparability. Root causes include (i) heterogeneous reference methods (BMD, VITEK 2, disk diffusion) and breakpoints (CLSI vs EUCAST); (ii) variable reading time points and ATU handling; and (iii) inconsistent error metrics and QC strains [30,31]. Without standardized protocols, common inoculum thresholds, defined reading windows, unified acceptance criteria, and shared QC strains results from different platforms cannot be reliably aligned, undermining both meta-comparison and clinical confidence [30,31]. Establishing harmonized protocols is therefore essential to ensure result reliability and cross-platform alignment.

### 3.2 Pre-analytical factors

RAST accuracy is sensitive to pre-analytical variables. EUCAST RAST specifies 125 μL ± 25 μL inoculation with strict reading at 4, 6, 8, and 16–20 h, and zones within the ATU accounted for 23.3% of non-interpretable measurements in one evaluation [18,19]; QuickMIC processing uses 10 μL of positive blood cultures with a 15-min agarose solidification step [23,24]. Blood-culture incubation time before testing, inoculum density thresholds, blood-culture bottle type (e.g., resin vs standard media), sample-processing delays, and blood-to-broth ratios all influence growth kinetics and zone reading. Agreement therefore varies by organism, bottle type, and antibiotic, and each laboratory must validate these conditions locally [18,20].

### 3.3 MDR detection errors and validation gaps

Persistent errors occur for MDR organisms, e.g., VITEK REVEAL showed ME 56.3% for trimethoprim–sulfamethoxazole and elevated errors for *NDM* producers [16], and QuickMIC showed a meropenem “I”-reporting bias [24]. Many microfluidic methods remain at the laboratory-validation stage [2], and molecular RAST cannot supply MICs [25,26]. For highly resistant populations, errors cluster around specific determinants (*NDM-5* for QuickMIC; *KPC* for EUCAST RAST), underscoring the need for mechanism-aware interpretation.

### 3.4 Clinical implementation considerations

Beyond analytical performance, adoption depends on economic, workflow, and regulatory realities. Direct costs differ sharply: EUCAST RAST has low consumable cost and is attractive for resource-limited and low- and middle-income country (LMIC) settings [18,19], whereas automated platforms (QuickMIC, VITEK REVEAL, Accelerate Pheno) require dedicated instruments with high acquisition and service costs that may limit implementation, particularly where the AMR mortality burden is greatest [13,16,22,23]. Workflow fit matters: manual RAST is labor-intensive at strict time points and benefits from total laboratory automation (e.g., WASPLab) [20], whereas fully automated systems reduce hands-on time but demand laboratory information system (LIS) integration.

High-volume academic centers with 24/7 staffing and automation can exploit the speed of instrumented platforms; community hospitals with limited shifts and budgets may favor low-cost EUCAST RAST (with extended 16–20 h incubation to accommodate operating hours) or single-platform automation. The choice should weigh test volume, staffing, existing infrastructure, and local resistance epidemiology.

VITEK REVEAL (CE-IVD, FDA 510(k)) [13], Accelerate PhenoTest BC (US-IVD/FDA-cleared) [22], QuickMIC (CE-marked) [23,24], BioFire BCID2 and Verigene (FDA-cleared/CE-marked) [25,26] are commercially available; uRAST and several single-cell/microfluidic concepts remain research-stage [2].

RAST implementation should follow defined quality control (QC) procedures. Reference QC strains such as *E. coli* ATCC 25922, *P. aeruginosa* ATCC 27853, and *S. aureus* ATCC 29213 should be used routinely at the frequency prescribed by CLSI/EUCAST, with acceptance monitored per standard AST QC [18]. For routine adoption as a sole method, the accepted criteria align with FDA AST requirements: CA ≥90.0%, minor error ≤10.0%, ME ≤ 3.0%, and VME ≤1.5% [2]. Agents that do not meet all thresholds require confirmation by an alternative method. EUCAST additionally publishes dedicated RAST Quality Control criteria (version 5.0, 2022) [18]. These criteria are essential for clinical validation, routine use, and accreditation compliance.

## 4 Future directions

RAST optimization will advance along several convergent axes: AI integration, point-of-care testing (POCT) deployment, and cross-disciplinary materials-science innovation. These three directions differ in technical maturity and clinical readiness. A concrete near-term application is AI-assisted automated result interpretation. Deep-learning models trained on growth-kinetics images or VOC sensor arrays can standardize zone/MIC calling, flag ATU results, and reduce inter-operator variability, thereby improving accuracy and efficiency while enabling decision support for antimicrobial stewardship. Among the three, this is the most immediately actionable direction. Miniaturized, capillary-driven microfluidic formats aligned with ISO 22870:2006 criteria, characterized by low reagent use, simplified workflow, and minimal operator input, are moving RAST toward bedside and resource-constrained settings and represent the nearer-term hardware path, although bedside deployment still requires field validation. Convergence with materials science is illustrated by aggregation-induced-emission (AIE) bioprobes for rapid phenotypic AST. Recent work demonstrates AIEgens for microorganism visualization and therapy [32], and a rapid AST of Gram-negative BSIs using an AIE bioprobe that “lights up” resistance [33], highlighting the potential of cross-disciplinary tools for next-generation RAST. However, AIE-based AST remains at the research/preclinical stage and belongs to the next generation of tools rather than near-term practice.

## CRediT authorship contribution statement

**Haiyi Li:** Writing – original draft. **Qiongfang Zhu:** Investigation. **Yalu Ren:** Investigation. **Meili Shen:** Writing – review & editing. **Jie Xu:** Writing – review & editing, Conceptualization.

## Funding

This work was supported by The Basic Research Special Project Foundation of Suzhou (No. SSD2024085) and the 10.13039/100008648Pfizer Foundation (No.76080151).

## Declaration of competing interest

The authors declare the following financial interests/personal relationships which may be considered as potential competing interests:MS was employed by Nanjing Dinfectome Technology Inc. The remaining authors declare that the research was conducted in the absence of any commercial or financial relationships that could be construed as a potential conflict of interest. If there are other authors, they declare that they have no known competing financial interests or personal relationships that could have appeared to influence the work reported in this paper.

## Data Availability

No data was used for the research described in the article.
