# Supplementary material for: Direct rapid antimicrobial susceptibility testing (RAST) from positive blood cultures: Recent advances, challenges, and future directions
Source: Pract Lab Med. 2026 Sep 4;51:e00555. doi: 10.1016/j.plabm.2026.e00555 (PMC13572009; doi:10.1016/j.plabm.2026.e00555)
Supplement: Supplementary file 1 [file mmc1.docx]

Author Checklist Clinica Chimica Acta (CCA)

Manuscript Title: Advances in Direct Rapid Antimicrobial Susceptibility Testing of Positive Blood Culture Bottles

Corresponding Author: Jie Xu

Date: March 23, 2026

1. **Scope:** Make sure your research topic is well within the scope of the CCA journal, which focuses on diagnostic laboratory medicine. Studies should focus on analysis of human body fluids (e.g., blood, serum/plasma, urine, cerebrospinal fluids, saliva, ascites, effusion fluids, amniotic fluid, gastric juice, sweat, faeces, expired air). Studies focused on analysis of tissue are considered as out of scope and will be desk rejected. For more information about the scope, see aims and scope on Science Direct: <https://www.sciencedirect.com/journal/clinica-chimica-acta>. Given that the journal publishes in Clinical Chemistry, the editors expect that at least 10% of the references in your manuscript originate from clinical laboratory medicine-oriented journals.

Confirmed – Within scope. A total of ≥15 references were sourced from journals related to Clinical Laboratory Medicine, accounting for ≥51.7% of the 29 total references, which exceeds the journal’s 10% minimum requirement.

**Please note that the editors are considering the following topics as OUT OF SCOPE.** Manuscripts dealing with these topics will typically be desk rejected:

a. **Systematic reviews and meta-analyses**. Systematic reviews and meta-analyses are typically not considered by CCA unless the methods are particularly rigorous and transparent (including a clear description of the search strategy, inclusion and exclusion criteria, and statistical methods used) AND the findings are of direct significance to laboratory medicine. Studies from a single country or region are typically not considered as they may not be generalizable to other populations.

This article is a narrative review, not a systematic review or meta-analysis. It did not employ a systematic literature search strategy, PRISMA flow, or statistical pooling analysis, and thus does not fall into the category of systematic reviews/meta-analyses explicitly deemed out of scope by Clinica Chimica Acta (CCA).

This review includes multicenter studies from multiple countries across Europe, North America, Asia, and other regions. The evaluated content demonstrates broad international applicability and is free from limitations associated with a single country or region.

b. **Studies focused on ratios of routinely measured markers** (e.g., neutrophil:lymphocyte or similar ratios, ratios with albumin, triglycerides, etc.). Such studies will not be considered. Refer to: Delanghe JR, Speeckaert MM, De Buyzere ML. On the use of lymphocyte to neutrophil ratios in laboratory medicine. Clin Chim Acta. 2020 Nov;510:26-27. doi: 10.1016/j.cca.2020.07.008. PMID: 32649954.

Not applicable. This article does not involve the analysis of conventional marker ratios such as the neutrophil-to-lymphocyte ratio or albumin ratio, and is therefore completely irrelevant to this exclusion criterion.

3. **Properly Cited Methods**: Ensure that all methods used in your research are properly cited and referenced, providing enough detail for readers to understand and replicate the experiments.

Confirmed. All detection methods involved in this review are accompanied by citations of the original literature, as detailed below:

- VITEK® REVEAL™ [3][10][14][15][16]
- EUCAST RAST [5][8][9][17][18][27]
- Accelerate PhenoTest BC [4][13][21]
- uRAST [2]
- BIOFIRE® BCID2 Panel [16][27]
- Verigene BC Test [4][15]
- QuickMIC [22]
- Impedance-sensing AST [23]
- Stimulated Raman Scattering [11]
- Modified VITEK 2 Compact RAST [6]
- ATP bioluminescence [29]

4. **Validated Methods:** Use validated methods and assays, especially when dealing with immunoassays or other diagnostic tests. Avoid using unvalidated or unreliable methods, as they may lead to inaccurate results and conclusions. If using assays that are not clinically approved from a regulatory body or not widely accepted, method validation data must be included in the manuscript or referenced as peer-reviewed in another publication(s).

The regulatory approval status of the main RAST platforms described in this article is as follows. For technologies still in the laboratory validation phase (e.g., impedance-sensing AST, uRAST), their developmental/validation status has been clearly labeled in the text, with corresponding validation data provided via citations of peer-reviewed literature [2][23].

| Platform | Regulatory Status | Notes |
| --- | --- | --- |
| VITEK® REVEAL™ | CE-IVD certification + FDA 510(k) clearance | Dual regulatory approvals, as explicitly stated in [10][14] |
| EUCAST RAST | EUCAST official recommended protocol (published 2019) | Multicenter validation across 55 European laboratories [8] |
| Accelerate PhenoTest BC | FDA approval | Multicenter clinical validation [4][13][21] |
| BIOFIRE® BCID2 Panel | FDA approval + CE-IVD | Cited in [16][27] |
| Verigene BC Test | FDA approval | Cited in [4][15] |

5. **Appropriate Use of eGFR Calculations**: Where relevant, use formula(s) for calculating the estimated glomerular filtration rate (eGFR) that are appropriate for the population being studied. Appropriate restrictions of the used formulas (e.g., age range, limit of quantitative expression, precision) should be respected.

Not applicable.

6. **Originality of Case Reports:** Case reports should focus on clinical laboratory results and their role in the diagnosis or case resolution. Submit only original case reports that have not been previously published or presented elsewhere. Ensure that your case report contributes new knowledge or insights to the field of laboratory medicine. Case reports that focus on clinical descriptions found with a genetic mutation are typically not considered.

Not applicable.

7. **Hardy-Weinberg Equilibrium Compliance:** When dealing with genetic data, ensure that your results of control populations are consistent with the Hardy-Weinberg equilibrium or provide a justified explanation for any deviations.

Not applicable.

8. **Adherence to Disclosure Rules:** Follow the journal's guidelines for disclosing any conflicts of interest, funding sources, or other relevant information that may influence the interpretation of your research results.

Confirmed – All disclosures complete.

9. **Statistics:** The use of SEM (standard error of the mean) is strongly discouraged. When dealing with non-Gaussian distributions, the appropriate non-parametric statistics should be used. When drawing conclusions regarding populations, be sure that the study is not statistically underpowered. It is advised to make a power calculation in order to obtain the required number of subjects to be enrolled in the study.

Confirmed – Not applicable to this review; data from cited studies reported as published.

10. **Candidate Reviewers:** Candidate reviewers must have an institutional e-mail address, not a generic e-mail address (e.g., Hotmail, Yahoo, Gmail, Outlook, AOL, etc.) and must have a different nationality from the authors.

We regret that we are currently unable to provide suggested reviewers for this submission. We would greatly appreciate it if the editorial office could arrange for suitable peer reviewers with relevant expertise in antimicrobial susceptibility testing and clinical microbiology.
